# Complete Genome Sequence of the Larval Shellfish Pathogen *Vibrio tubiashii* Type Strain ATCC 19109

**DOI:** 10.1128/genomeA.01252-14

**Published:** 2014-12-18

**Authors:** Gary P. Richards, David S. Needleman, Michael A. Watson, James L. Bono

**Affiliations:** a U.S. Department of Agriculture, Agricultural Research Service, Dover, Delaware, USA; b U.S. Department of Agriculture, Agricultural Research Service, Wyndmoor, Pennsylvania, USA; c U.S. Department of Agriculture, Agricultural Research Service, Clay Center, Nebraska, USA

## Abstract

*Vibrio tubiashii* is a larval shellfish pathogen. Here, we report the first closed genome sequence for this species (ATCC type strain 19109), which consists of two chromosomes (3,294,490 and 1,766,582 bp), two megaplasmids (251,408 and 122,808 bp), and two plasmids (57,076 and 47,973 bp).

## GENOME ANNOUNCEMENT

*Vibrio tubiashii* is a naturally occurring, Gram-negative, marine bacterium that has been associated with high mortalities of larval shellfish (1–4) and significant economic losses in shellfish hatcheries (5) leading to reductions in the availability of seed oysters and clams needed for commercial shellfish planting. *Vibrio tubiashii* causes bacillary necrosis, first recognized by Tubiash et al. in 1965 (1). Although *V. tubiashii* has been known for years, the lack of a complete genome sequence has slowed the pace of research on this pathogen. Much confusion has also occurred because of the misidentification of some *V. tubiashii* strains; strains thought to be *V. tubiashii*, like ATCC 19105, which was later identified as the shellfish pathogen *V. coralliilyticus* (4, 6, 7). The misidentification of these pathogens has complicated the discernment of the roles they play in larval shellfish mortalities.

The type strain for *V. tubiashii* is ATCC 19109 and was sequenced using a PacBio RS II system (Pacific Biosciences, Menlo Park, CA) on single-molecule real-time (SMRT) cells using PacBio P5-C3 chemistry. Subread filtering was performed with the SMRT Analysis Software suite (8), error correction and assembly was conducted with Celera Assembler v8.1 (9), overlapping ends were trimmed using Geneious v7.1.5 (Biomatters, Auckland, New Zealand) and polished with Quiver (8). Coverage was 20× and assemblies gave a consensus accuracy of 99.9996 to 100%. The fully assembled genome contains two closed chromosomes, two closed megaplasmids (p251 and p123), and two closed plasmids (p57 and p48). The genome contains a total of 5,540,337 bp consisting of chromosome 1 (3,294,490 bp), chromosome 2 (1,766,582 bp), and plasmids p251 (251,408 bp), p123 (122,808 bp), p57 (57,076 bp) and p48 (47,973 bp). This is the first complete genome sequence reported for any *V. tubiashii* strain.

Genome annotation for *V. tubiashii* ATCC 19109 was acquired from the NCBI Prokaryotic Genome Annotation Pipeline (Bethesda, MD) and revealed 5,080 genes, 4,918 coding sequences, 12 pseudogenes, 31 rRNAs (5S, 16S, and 23S), 117 tRNAs, 2 noncoding RNAs, and 7 frameshift genes. Together, these genomic and plasmid sequences are important references for the identification and comparison of potential virulence genes for this type strain and for other strains within this species.

### Nucleotide sequence accession numbers.

The complete genomic sequence of *V. tubiashii* ATCC 19109 (chromosomes 1 and 2 and the four plasmids) has been deposited in GenBank under accession no. CP009354, CP009355, CP009356, CP009357, CP009358, and CP009359.
